# Effects of combined aerobic and resistance training on gut microbiota and cardiovascular risk factors in physically active elderly women: A randomized controlled trial

**DOI:** 10.3389/fphys.2022.1004863

**Published:** 2022-10-20

**Authors:** Fei Zhong, Yongjin Xu, Hsin-Yi Lai, Min Yang, Lei Cheng, Xinger Liu, Xiaomin Sun, Yi Yang, Jian Wang, Wen Lv, Cong Huang

**Affiliations:** ^1^ Department of Sports and Exercise Science, Zhejiang University, Hangzhou, China; ^2^ Department of Neurology and Research Center of Neurology in Second Affiliated Hospital, Key Laboratory of Medical Neurobiology of Zhejiang Province, and Interdisciplinary Institute of Neuroscience and Technology, Zhejiang University School of Medicine, Hangzhou, China; ^3^ Key Laboratory for Biomedical Engineering of Ministry of Education, College of Biomedical Engineering and Instrument Science, Zhejiang University School of Medicine, Hangzhou, China; ^4^ Department of Neurology of Sir Run Run Shaw Hospital, Zhejiang University School of Medicine, Hangzhou, China; ^5^ School of Public Health, Zhejiang University, Hangzhou, China; ^6^ MOE Key Laboratory of Biosystems Homeostasis and Protection, College of Life Sciences, Zhejiang University, Hangzhou, China; ^7^ Kunshan Old Companion Home Care Service Agency, Kunshan, China; ^8^ Department of Nutrition and Food Safety, School of Public Health, Xi’an Jiaotong University Health Science Center, Xi’an, China; ^9^ Global Health Institute, Xi’an Jiaotong University Health Science Center, Xi’an, China; ^10^ Faculty of Sport Sciences, Waseda University, Saitama, Japan; ^11^ Center for Psychological Sciences, Zhejiang University, Hangzhou, China; ^12^ Department of Medicine and Science in Sports and Exercise, Tohoku University Graduate School of Medicine, Sendai, Japan

**Keywords:** physical function, sarcopenic obesity, metabolic syndrome, gut microbiota, elderly adults, randomized control trial

## Abstract

**Background:** Exercise can modulate gut microbiota and lower the risk of cardiovascular disease (CVD). However, the association between exercise-induced changes in gut microbiota and CVD risk have not been investigated.

**Objective:** This study determined the effects of exercise training on CVD risk and gut microbiota in physically active elderly women and whether exercise-induced gut microbiota changes were associated with CVD risk.

**Methods:** An 8-week randomized controlled trial was conducted with 14 elderly women assigned to exercise group (*n* = 8) or control group (*n* = 6). Physical function, sarcopenic obesity, and metabolic syndrome were evaluated as components of CVD risk. Gut microbiota composition was determined using 16S rRNA gene sequencing. Repeated-measures analysis of variance was used to examine intra-group and inter-group differences.

**Results:** A significant group × time interaction was observed for chair sit-and-reach (F = 8.262, *p* = 0.014), single-leg standing with eyes closed (F = 7.340, *p* = 0.019), waist circumference (F = 6.254, *p* = 0.028), and body fat mass (F = 12.263, *p* = 0.004), for which the exercise group showed improved trends. The exercise group exhibited significant improvements in skeletal muscle mass (*p* = 0.041) and fasting blood glucose (*p* = 0.017). Regarding gut microbiota, a significant interaction was observed for the class Betaproteobacteria (F = 6.822, *p* = 0.023) and genus *Holdemania* (F = 4.852, *p* = 0.048).

**Conclusion:** The 8-week exercise training improved physical function, lowered CVD risk, and modulated relative abundance of gut microbiota associated with CVD in physically active elderly women.

## 1 Introduction

Cardiovascular disease (CVD) is characterized by pathologic changes of the heart, blood vessels and cerebrovascular system (Cannon, 2013), which is the major cause of death globally, accounting for approximately 17.8 million deaths in 2017 (Kyu et al., 2018). The prevalence of CVD is high in the elderly Chinese population and there is an increasing trend in the younger population (Wang et al., 2015). Previous studies have reported that multiple risk factors contribute to the development of CVD such as metabolic syndrome (Lakka et al., 2002), sarcopenia (Matsubara et al., 2017). Among these aspects, physical inactivity is an important factor in the induction of CVD, which is also the modifiable behavioral risk factor most strongly associated with CVD except for tobacco use (Yusuf et al., 2020). Conversely, regular exercise can have a favorable effect on many risk factors for CVD (Thompson et al., 2003). However, the effects of the type and volume of exercise on CVD risk requires additional attention.

A recent study showed that combined aerobic and resistance exercise may provide more comprehensive benefits and a greater reduction of CVD risk compared to that of aerobic or resistance exercise alone (Schroeder et al., 2019). Moreover, randomized controlled trial studies of exercise-induced cardiovascular risk reduction have focused on physically inactive older adults (Beavers et al., 2018). This has led to a lack of effective evidence regarding people with habits of physical activity. The significance of this is highlighted by the fact that a dose–response association was observed between physical activity level and risk of CVD in a recent large cohort study (Ramakrishnan et al., 2021). The association was found whether total physical activity level, moderate-intensity physical activity level, or vigorous-intensity physical activity level was considered. Thus, it is of interest and relevance to determine the effect of combined aerobic and resistance exercise on CVD risk in elderly people that maintain exercise habits.

The potential mechanism of aerobic exercise-induced CVD risk reduction is *via* a pathway that may lead to increased insulin sensitivity (Gao et al., 2002), while resistance exercise may regulate mitophagy and inhibiting oxidative stress (Li et al., 2021). However, deeper potential mechanisms need to be explored. Interestingly, the effects of changes in the gut microbiota composition on CVD risk has attracted attention and gut microbiota dysbiosis may contribute to a higher risk of CVD (Witkowski et al., 2020). Furthermore, accumulating evidence demonstrates that exercise can influence the gut microbiota composition in humans. We previously reported that an 8-week exercise training regimen can induce the reduction of bacteria associated with pro-inflammation, such as Proteobacteria, in physically inactive older women (Zhong et al., 2020). The underlying mechanisms that exercise-induced changes of gut microbiota involved the acceleration of the mixing of internal contents (Mailing et al., 2019) and colon transit (Song et al., 2012), and the improvement of the permeability of the intestinal mucosal barrier (Holland et al., 2015). Furthermore, exercise can regulate the hypothalamic–pituitary–adrenal (HPA) axis, thereby affecting the composition of the gut microbiota (Bermon et al., 2015). However, little is currently known regarding the association between exercise-induced change in gut microbiota composition and CVD risk.

Our current study investigated the effects of an 8-week combined aerobic and resistance training regimen on CVD risk and gut microbiota composition in physically active elderly women. We also aimed to determine whether exercise-induced changes in the composition of gut microbiota were associated with CVD risk.

## 2 Materials and methods

### 2.1 Study design

An 8-week RCT of combined aerobic and resistance training was performed. The study was conducted from December 2020 to February 2021. The study protocol was approved by the Ethics Committee at the School of Public Health Zhejiang University (Code: EGL201810-2) and registered in the Chinese Clinical Trial Registry (Code: ChiCTR1800019711). All study participants provided written informed consent.

### 2.2 Inclusion and exclusion criteria

The participants were recruited from the community and of the age between 60 and 75 years (inclusive). These participants were capable of living independently. Participants were excluded for the following reasons: 1) had been treated for a malignant tumor; 2) had experienced acute myocardial infarction, heart failure; 3) had a history of stroke; 4) was taking antihypertensive or lipid lowering medications; 5) had any disease that restricted physical activity; 6) had any medical condition that limited the ability to perform safe exercise; 7) presented with fasting triglyceride ≥2.26 mmol/L, serum creatinine ≥200 μmol/L, systolic blood pressure ≥160 mmHg, or diastolic blood pressure ≥100 mmHg; 8) had some degree of cognitive impairment with a Mini-Mental State Examination (MMSE) score of less than 24; 9) had taken antibiotics within 1 month prior to the start of the study.

**FIGURE 1:**
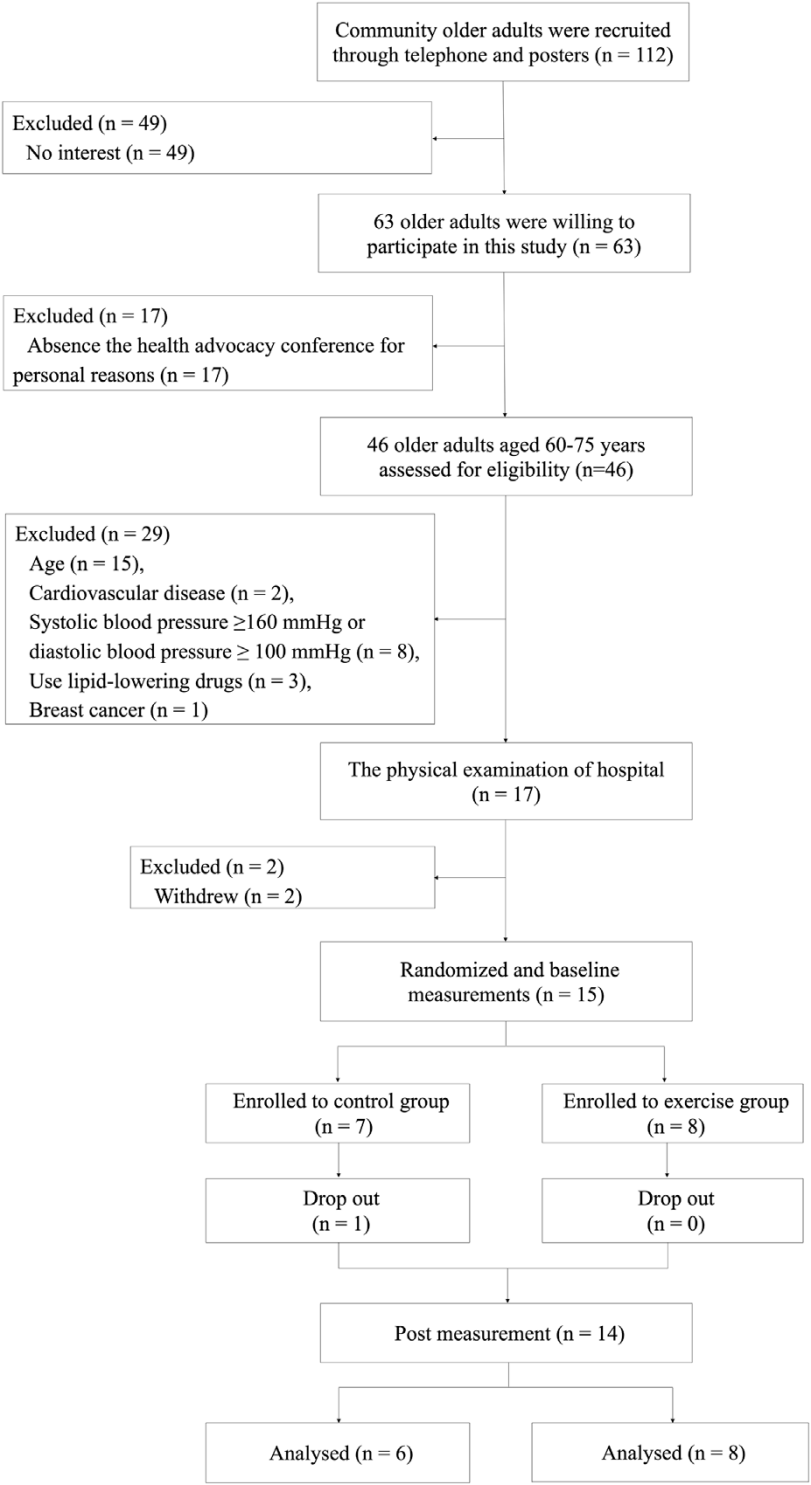
Fow diagram of the research design of the randomized controlled trial. Recruitment, screening, randomization, intervention, outcomes assessment, and data analysis.

A total of 112 older adults were recruited in the community through telephone and posters, of which 63 were willing to participate in this study, and 46 of them attended the health advocacy conference on time. Individuals were selected *via* a questionnaire and health examination. 29 subjects were excluded: 15 individuals did not meet the age requirements, 2 individuals had CVD, 8 individuals had hypertension, 3 individuals had hyperlipidemia, 1 individual was a breast cancer patient. 2 people withdrew prior to the hospital-based medical examination. Ultimately, 15 participants were randomly assigned to the Exe group (*n* = 8) and Con group (*n* = 7). A computer-generated list of random numbers was used to assign participants to the groups. After the intervention, one participant assigned to the Con group dropped out of the study. Therefore, the final analysis included eight subjects in the Exe group and six subjects in the Con group. The study design of the trial is shown in Figure 1.

### 2.3 Exercise intervention

Participants assigned to the Con group were to maintain daily life. The subjects in the Exe group took part in exercise program in which they met four times per week from 9:30 am to 10:30 am. Each session lasted approximately 60 min, including a warm-up period (10 min), aerobic exercise (20 min), resistance exercise (25 min), and a cool down period (5 min). The protocols of the exercise intervention have been described previously (Zhong et al., 2020).

### 2.4 Demographic and anthropometric parameters

Information regarding socio-demographics (age, sex, monthly income, educational level, marital status, residence status), health-related behaviors (smoking status, alcohol use), and physical activity were collected. Individuals with a Physical Activity Scale for the Elderly (PASE) score greater than 90 tend to undergo moderate to intense activity in their daily life (Curcio et al., 2019). A standardized protocol was used to assess body height (cm) and weight (kg). BMI was calculated [weight (kg)/height squared (m^2^)].

Chair sit-and-reach, single-leg standing with eyes closed, 2-minute step test, grip strength, 30-s chair stand test, and timed up and go test were used to assess flexibility, balance, aerobic capacity, muscle strength, and functional mobility of the participants (Podsiadlo and Richardson, 1991; Rikli and Jones, 1999). A V-BODY HBF-701 bioelectrical impedance analyzer (Omron Healthcare Co. Ltd., Kyoto, Japan) was used to estimate the percentage of skeletal muscle mass. Skeletal muscle mass (kg) was calculated as body weight (kg) × skeletal muscle percentage and body fat (kg) was calculated as body weight (kg) × body fat percentage (Ohara et al., 2014; Ohara et al., 2015).

### 2.5 Stool sample collection and genomic DNA extraction

Stool samples of the participants were collected before the baseline test and after the eight weeks of exercise training. A MagPure Stool DNA KF Kit B (Magen, China) was used to extract microbial community DNA. The 16S rRNA gene of the gut microbiota was amplified using degenerate PCR primers of regions V3–V4. Sequencing was performed using the Illumina MiSeq platform (BGI, Shenzhen, China) and 2 × 300 bp paired-end reads were generated. After filtering the raw reads, the Fast Length Adjustment of SHort reads (FLASH, v1.2.11) software was used for adding the paired-end reads to tags (Magoc and Salzberg, 2011). The UPARSE (USEARCH version 7.0.1090) software (Edgar, 2013) was used to cluster the tags into operational taxonomic units (OTUs) and UCHIME (v4.2.40) (Edgar et al., 2011) was used to detect chimera sequences. The OTU representative sequences were classified using Ribosomal Database Project (RDP) Classifier v.2.2 and trained using QIIME v1.8.0 software (Caporaso et al., 2010). USEARCH_global was used to compare tags back to the OTUs and then OTU abundance was obtain for each sample (Edgar, 2010).

### 2.6 Statistical analyses

SPSS Statistics version 23.0 software (SPSS Inc., Chicago, IL, United States) was used to analyze the data. Mean ± standard deviation is used to describe continuous variables and percentage is used to expressed categorical variables. Normal distribution of the variables was verified using the Shapiro-Wilk test. The independent samples *t*-test (normally distributed data) and Mann-Whitney *U* test (non-normally distributed data) were used for the comparison between two groups. Comparisons of categorical variables were estimated using Fisher’s exact test. Paired samples *t*-test (normally distributed data) and Wilcoxon signed-ranks test (non-normally distributed data) were used for comparisons within groups. Repeated-measures analysis of variance (RM-ANOVA) was used to examine intra-group and inter-group differences of the main observational variables. A 95% confidence interval (CI) was selected to calculate differences between groups, there was statistical differences when *p* < 0.05. The association between two variables was evaluated using Pearson correlation analysis.

## 3 Results

### 3.1 Baseline characteristics of the study subjects

Baseline characteristics of the study participants are shown in Table 1. At baseline, there were no significant differences in demographic or anthropometric parameters between the two groups. The age of the participants in the Exe group was 66.38 ± 4.07 years and 68.50 ± 3.78 years in the Con group. The average height of the Exe group was 157.94 ± 7.07 cm compared to 156.58 ± 5.30 cm for the Con group. The average weight was 52.85 ± 6.82 kg for the Exe group and 52.88 ± 2.90 kg for the Con group. The body mass index (BMI) for the Exe group was 21.16 ± 2.17 kg/m^2^ compared to 21.61 ± 1.50 kg/m^2^ for the Con group. Physical Activity Scale for the Elderly (PASE) scores for the Exe group was 117.11 ± 24.49 and 105.36 ± 49.90 for the Con group. Therefore, the participants of our study were physically active elderly women. Finally, no statistical differences were detected in education, marital status, residential status, smoking, or alcohol use between the two study groups.

**TABLE 1:** Demographic characteristics of the participants at baseline.

Characteristics	Exercise group (n = 8)	Control group (n = 6)	Difference between groups (*p*)
Female, n (%)	8 (100)	6 (100)	-
Age (y)	66.38 ± 4.07	68.50 ± 3.78	0.339
Height (cm)	157.94 ± 7.07	156.58 ± 5.30	0.702
Weight (kg)	52.85 ± 6.82	52.88 ± 2.90	0.990
BMI (kg/m^2^)	21.16 ± 2.17	21.61 ± 1.50	0.675
Monthly income (RMB)	2937.50 ± 1953.71	2900.00 ± 1002.00	0.967
PASE scores	117.11 ± 24.49	105.36 ± 49.90	0.612
Education, n (%)			
<Senior high school	5 (62.5)	2 (33.3)	0.592
≥Senior high school	3 (37.5)	4 (66.7)	
Marital status, n (%)			
Married	8 (100)	4 (66.7)	0.165
Divorced or widowed	0	2 (33.3)	
Residential status, n (%)			
Live alone	2 (25)	1 (16.7)	1.000
Not live alone	6 (75)	5 (83.3)	
Supporting sources, n (%)			
Pension	7 (87.5)	5 (83.3)	0.692
Children’s support	1 (12.5)	0 (0)	
National minimum subsistence allowance	0 (0)	1 (16.7)	
Number of smokers, n (%)			
Non-smoking, n (%)	8 (100)	6 (100)	-
Alcohol use, n (%)			
Not drinking	7 (87.5)	6 (100)	1.000
Drinking now	1 (12.5)	0 (0)	

Data of continuous variables are expressed as mean ± standard deviation; Data of categorical variables are described as number and percentages.

### 3.2 Changes in microbiota diversity and species composition following the intervention

**FIGURE 2:**
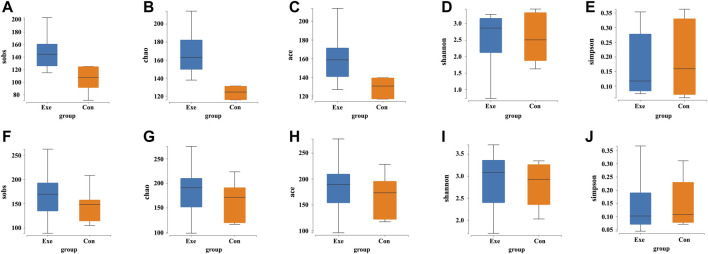
Changes in the alpha diversity indices of gut microbiota after exercise training. Exe: combined aerobic and resistance exercise, Con: maintaining daily life; sobs, observed species index; chao, Chao index; ace, Ace index; shannon, Shannon index; simpson, Simpson index; **(A–E)** Pre-exercise. **(F–J)** After 8-week exercise regimen.

Significant increases were found in alpha diversity of the Sobs index (*p* = 0.008), Chao index (*p* = 0.017), and Ace index (*p* = 0.002) in the Exe group after intervention (Supplementary Table S1; Figure 2). A significant time main effect also was observed for these three variables [Sobs (F = 15.273, *p* = 0.002); Chao (F = 13.402, *p* = 0.003); Ace (F = 16.637, *p* = 0.002)].

**FIGURE 3:**
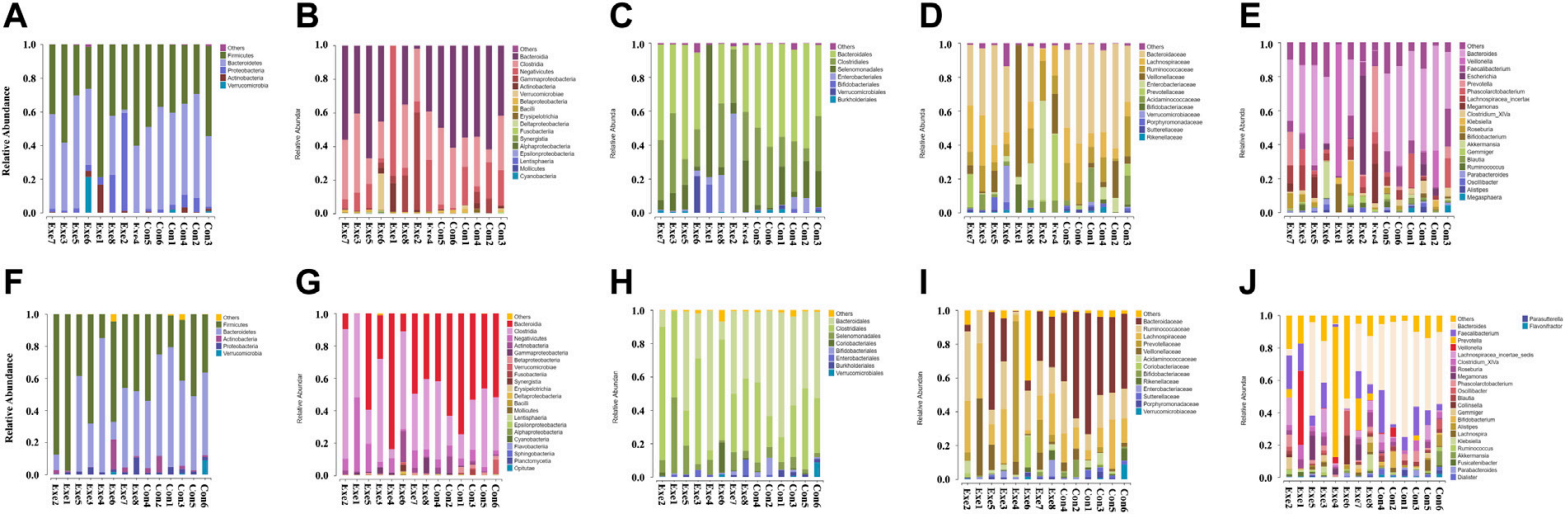
Changes in the relative abundance of gut microbiota components after exercise training. Exe: combined aerobic and resistance exercise, Con: maintaining daily life. **(A–E)** Pre-exercise. **(F–J)** After 8-week exercise regimen. **(A,F)** Phylum; **(B,G)** Class; **(C,H)** Order; **(D,I)** Family; **(E,J)** Genus.

At the class, order, and family level, a significant group × time interaction was observed for the relative abundance of Betaproteobacteria (F = 6.822, *p* = 0.023), and Sutterellaceae (F = 7.075, *p* = 0.021), respectively (Table 2) and (Figure 3). These bacteria in the Con group showed a significant simple effect of group. Additionally, there was a significant group × time interaction for the genus *Holdemania* (F = 4.852, *p* = 0.048) and a significant simple effect of group was demonstrated in the Exe group (F = 6.055, *p* = 0.030).

**TABLE 2:** Changes in gut microbiota and cardiovascular risk factors in each group following the intervention.

		Level	Specific	Exercise group (n = 8)			Control group (n = 6)			Main effect (Group × Time)		Simple effect (time)				Simple effect (group)			
				Baseline	After intervention	*p*	Baseline	After intervention	*p*	F	*p*	time	F	*p*	Mean difference (95% CI)	group	F	*p*	Mean difference
Gut microbiota taxa		Class	Betaproteobacteria	0.50 ± 0.55	0.45 ± 0.44	0.313	1.05 ± 0.96	1.63 ± 1.48	0.089	6.822	0.023	Pre	1.871	0.196	−0.553 (−1.434, 0.328)	exe	0.101	0.756	−0.051 (−0.398, 0.296)
												post	4.721	0.051	−1.189 (−2.380, 0.003)	con	10.113	0.008	0.585 (0.184, 0.986)
		Order	Burkholderiales	0.50 ± 0.55	0.45 ± 0.44	0.319	1.05 ± 0.96	1.63 ± 1.48	0.089	6.829	0.023	Pre	1.869	0.197	−0.553 (−1.434, 0.328)	exe	0.098	0.760	−0.050 (−0.397, 0.297)
												post	4.721	0.051	−1.189 (−2.380, 0.003)	con	10.152	0.008	0.586 (0.185, 0.987)
		Family	Sutterellaceae	0.50 ± 0.55	0.44 ± 0.45	0.226	1.05 ± 0.96	1.63 ± 1.48	0.089	7.075	0.021	Pre	1.869	0.197	−0.553 (−1.434, 0.328.)	exe	0.145	0.710	−0.061 (−0.407, 0.286)
												post	4.784	0.049	−1.199 (−2.394, −0.005)	con	10.172	0.008	0.586 (0.186, 0.986)
		Genus	Holdemania	0.009 ± 0.0009	0.006 ± 0.006	0.075	0.015 ± 0.018	0.016 ± 0.019	0.324	4.852	0.048	Pre	0.726	0.411	−0.006 (−0.022, 0.010)	exe	6.055	0.030	−0.003 (−0.006, 0.000)
												post	2.158	0.168	−0.010 (−0.025, 0.005)	con	0.613	0.449	0.001 (−0.002, 0.004)
Cardiovascular risk factors	Sarcopenic obesity parameters	Physical function	Chair sit-and-reach (cm)	10.00 ± 6.55	19.06 ± 9.49	0.002	8.58 ± 5.50	9.50 ± 7.74	0.693	8.262	0.014	Pre	0.183	0.676	1.417 (−5.799, 8.632)	exe	23.861	0.0004	9.063 (5.020, 13.105)
												post	4.047	0.067	9.563 (−0.795, 19.920)	con	0.183	0.676	0.917 (−3.751, 5.584)
			Single leg standing with eyes closed (s)	7.67 ± 4.22	11.86 ± 7.77	0.046	6.88 ± 1.96	5.16 ± 1.87	0.131	7.340	0.019	Pre	0.179	0.680	0.790 (−3.282, 4.863)	exe	8.589	0.013	4.190 (1.075, 7.305)
												post	4.203	0.063	6.707 (−0.421, 13.836)	con	1.094	0.316	−1.727 (−5.324, 1.870)
		Anthropometry parameters	Waist circumference (cm)	78.25 ± 7.99	77.25 ± 6.36	0.364	78.30 ± 6.25	83.00 ± 7.56	0.093	6.254	0.028	Pre	0.0002	0.990	−0.050 (−8.657, 8.557)	exe	0.449	0.515	−1.000 (−4.251, 2.251)
												post	2.389	0.148	−5.750 (−13.856, 2.356)	con	7.441	0.018	4.700 (0.946, 8.454)
			Waist-hip ratio	0.82 ± 0.06	0.82 ± 0.04	0.975	0.82 ± 0.06	0.88 ± 0.07	0.040	5.935	0.031	Pre	0.002	0.961	−0.002 (−0.071, 0.068)	exe	0.001	0.978	0.000 (−0.033, 0.032)
												post	4.064	0.067	−0.056 (−0.118, 0.005)	con	10.226	0.008	0.054 (0.017, 0.092)
			Body fat mass (kg)	18.17 ± 3.33	17.45 ± 3.56	0.013	18.32 ± 3.14	18.93 ± 2.84	0.125	12.263	0.004	Pre	0.007	0.934	−0.149 (−3.980, 3.682)	exe	8.392	0.013	−0.718 (−1.259, −0.178)
												post	0.692	0.422	−1.475 (−5.338, 2.388)	con	4.510	0.055	0.608 (−0.016, 1.232)
	-	Metabolic parameters	COR (ug/dL)	10.03 ± 1.60	9.07 ± 1.59	0.069	8.28 ± 2.14	9.34 ± 2.81	0.066	7.084	0.021	Pre	3.106	0.103	1.754 (−0.414, 3.922)	exe	3.709	0.078	−0.960 (−2.046, 0.126)
												post	0.054	0.821	−0.273 (−2.841, 2.295)	con	3.434	0.089	1.067 (−0.187, 2.321)

Baseline: pre-exercise; After intervention: after 8-week exercise. Paired samples t-test was used to indicated intra-group differences of gut microbiota for data with normal distributions or Wilcoxon signed-ranks test for data with non-normal distributions. The group × time interaction of changes in gut microbiota composition was estimated by repeated-measures analysis of variance after 8-week exercise training. COR: cortisol.

The relative abundance of members of the order Coriobacteriales (*p* = 0.012) and family Coriobacteriaceae (*p* = 0.012) were significantly increased in the Exe group. We also observed a significant increase in the relative abundance of *Asaccharobacter* (*p* = 0.028), *Collinsella* (*p* = 0.028), and *Fusicatenibacter* (*p* = 0.049) in the Exe group and a significant time main effect was demonstrated for *Asaccharobacter* (F = 4.875, *p* = 0.047) (Supplementary Table S1; Figure 3). Furthermore, a significant main effect of group was detected for the relative abundance of members of the class Bacilli (F = 6.336, *p* = 0.027), order Lactobacillales (F = 6.378, *p* = 0.027), family Bacteroidaceae (F = 9.711, *p* = 0.009), genera *Anaerostipes* (F = 10.160, *p* = 0.008) and *Bacteroides* (F = 9.711, *p* = 0.009) (Supplementary Table S1) and (Figure 3).

### 3.3 Changes in cardiovascular risk factors following exercise intervention

#### 3.3.1 Changes in sarcopenic obesity components between the study groups

The physical function parameters of the subjects were evaluated, which included chair sit-and-reach, single-leg standing with eyes closed, 2-min step test, grip strength, 30-s chair stand test, and timed up and go test (Table 3). A significant group × time interaction was observed between the two groups for chair sit-and-reach (F = 8.262, *p* = 0.014) and single-leg standing with eyes closed (F = 7.340, *p* = 0.019). Both these physical functions in the Exe group were significantly improved compared to that in the Con group after exercise intervention [chair sit-and-reach (*p* = 0.002); single-leg standing with eyes closed (*p* = 0.046)]. The 2-min step test and grip strength scores did not change significantly, but did show a trend of improvement in the Exe group compared with that in the Con group.

**TABLE 3:** Changes in cardiovascular risk factors in each group following the intervention.

		Level	Specific	Exercise group (n = 8)			Control group (n = 6)			Main effect (time)			Main effect (group)			Interaction (Group × Time)	
				Baseline	After intervention	*p*	Baseline	After intervention	*p*	F	*p*	Mean difference	F	*p*	Mean difference	F	*p*
												(95% CI)			(95% CI)		
Cardiovascular risk factors	Sarcopenic obesity parameters	Physical function	2-min step test (counts)	97.50 ± 18.81	102.63 ± 14.32	0.286	84.50 ± 7.56	85.83 ± 18.07	0.883	0.513	0.488	3.229 (−6.596, 13.054)	4.392	0.058	14.896 (−0.591, 30.382)	0.177	0.682
			Grip strength (kg)	21.28 ± 5.90	21.40 ± 6.01	0.865	22.28 ± 4.97	20.47 ± 4.36	0.092	2.311	0.154	−0.846 (−2.058, 0.366)	0.000	0.990	−0.038 (−6.343, 6.268)	3.045	0.107
			Grip strength/weight	0.399 ± 0.077	0.403 ± 0.080	0.763	0.423 ± 0.099	0.382 ± 0.078	0.079	2.762	0.122	−0.018 (−0.043, 0.006)	0.001	0.979	−0.001 (−0.096, 0.093)	4.147	0.064
			30-second chair stand test (counts)	19.50 ± 1.77	18.25 ± 1.98	0.292	17.17 ± 2.71	16.17 ± 2.32	0.403	2.012	0.181	−1.125 (−2.853, 0.603)	6.534	0.025	2.208 (0.326, 4.091)	0.025	0.877
			Timed up and go test (s)	5.93 ± 0.40	5.79 ± 0.94	0.642	6.61 ± 1.06	6.59 ± 0.79	0.904	0.194	0.667	−0.080 (−0.477, 0.316)	3.328	0.093	−0.733 (−1.608, 0.142)	0.103	0.753
		Anthropometry parameters	Hip circumference (cm)	95.10 ± 4.55	93.94 ± 3.86	0.063	95.12 ± 3.11	94.50 ± 3.39	0.672	1.811	0.203	−0.890 (−2.330, 0.551)	0.022	0.885	1.968 (−4.579, 3.999)	0.170	0.687
			Weight (kg)	52.85 ± 6.82	52.74 ± 6.87	0.616	52.88 ± 2.90	53.62 ± 3.44	0.249	1.317	0.274	0.310 (−0.279, 0.900)	0.023	0.882	−0.456 (−7.039, 6.127)	2.444	0.144
			Skeletal muscle mass (kg)	12.16 ± 2.58	12.42 ± 2.49	0.041	11.97 ± 1.45	12.03 ± 1.79	0.739	2.670	0.128	0.163 (−0.054, 0.379)	0.057	0.815	0.284 (−2.300, 2.868)	0.957	0.347
	Metabolic syndrome parameters	-	BMI (kg/m^2^)	21.16 ± 2.17	21.26 ± 2.26	0.501	21.61 ± 1.50	21.60 ± 1.39	0.985	0.059	0.813	0.044 (−0.355, 0.444)	0.146	0.709	−0.392 (−2.629, 1.844)	0.081	0.780
			GLU (mmol/L)	5.23 ± 1.70	4.58 ± 1.48	0.017	4.69 ± 0.51	4.52 ± 0.52	0.169	12.551	0.004	−0.411 (−0.663, −0.158)	0.204	0.660	0.304 (−1.162, 1.769)	4.254	0.062
			SBP (mm Hg)	135.38 ± 21.90	132.88 ± 12.63	0.690	126.33 ± 14.88	131.17 ± 16.33	0.364	0.082	0.779	1.167 (−7.706, 10.039)	0.428	0.525	5.375 (−12.516, 23.266)	0.811	0.386
			DBP (mm Hg)	78.13 ± 12.74	80.25 ± 13.49	0.653	71.67 ± 9.35	73.17 ± 10.34	0.573	0.406	0.536	1.813 (−4.387, 8.012)	1.389	0.261	6.771 (−5.748, 19.289)	0.012	0.914
			TG (mmol/L)	1.30 ± 0.47	1.33 ± 0.31	0.824	0.95 ± 0.19	1.28 ± 0.38	0.080	3.960	0.070	0.173 (−0.016, 0.362)	1.304	0.276	0.201 (−0.183, 0.586)	2.946	0.112
			HDL-C (mmol/L)	1.90 ± 0.30	1.53 ± 0.26	<0.001	1.82 ± 0.22	1.58 ± 0.15	0.023	46.862	<0.001	−0.309 (−0.407, −0.210)	0.026	0.875	0.020 (−0.254, 0.294)	2.093	0.174
	Other metabolic parameters	-	CRP (mg/L)	2.63 ± 1.83	2.03 ± 1.03	0.389	2.20 ± 1.36	1.77 ± 0.50	0.324	2.040	0.179	−0.517 (−1.305, 0.271)	0.315	0.585	0.342 (−0.984, 1.667)	0.053	0.822
			Cr (μmol/L)	59.75 ± 8.55	68.25 ± 10.47	0.011	52.67 ± 7.58	62.83 ± 9.00	<0.001	214.504	<0.001	9.333 (7.945, 10.722)	1.659	0.222	6.250 (−4.323, 16.823)	1.710	0.215
			Urea (mmol/L)	5.43 ± 1.11	5.17 ± 0.94	0.237	5.31 ± 0.57	5.45 ± 0.71	0.550	0.167	0.690	−0.061 (−0.386, 0.264)	0.031	0.864	−0.080 (−1.074, 0.914)	1.786	0.206
			UA (μmol/L)	293.88 ± 66.87	293.25 ± 66.19	0.963	275.67 ± 36.35	258.33 ± 32.96	0.280	0.859	0.372	−8.979 (−30.084, 12.126)	0.876	0.368	26.563 (−35.277, 88.402)	0.744	0.405
			TC (mmol/L)	5.38 ± 1.12	5.52 ± 0.98	0.352	5.57 ± 0.38	6.01 ± 0.81	0.080	5.981	0.031	0.286 (0.031, 0.541)	0.533	0.479	−0.344 (−1.372, 0.683)	1.586	0.232
			LDL-C (mmol/L)	2.95 ± 0.85	2.73 ± 0.69	0.093	3.34 ± 0.35	3.18 ± 0.63	0.275	4.630	0.052	−0.190 (−0.383, 0.002)	1.418	0.257	−0.422 (−1.195, 0.350)	0.104	0.752
			HbA1c (%)	5.74 ± 1.21	5.65 ± 0.80	0.713	5.37 ± 0.29	5.38 ± 0.32	0.822	0.152	0.703	−0.035 (−0.233, 0.162)	0.560	0.469	0.319 (−0.610, 1.247)	0.329	0.577
			TNF-α (pg/ml)	6.79 ± 2.02	4.48 ± 2.91	0.005	6.93 ± 2.42	3.82 ± 3.11	0.007	36.730	<0.001	−2.715 (−3.690, −1.739)	0.036	0.852	0.256 (−2.676, 3.188)	0.806	0.387

Baseline: pre-exercise; After intervention: after 8-week exercise. Paired samples t-test was used to indicated intra-group differences of gut microbiota for data with normal distributions or Wilcoxon signed-ranks test for data with non-normal distributions. The group × time interaction of changes in CVD risk was estimated by repeated-measures analysis of variance after 8-week exercise training. BMI: body mass index; GLU: fasting blood glucose; SBP: systolic blood pressure; DBP: diastolic blood pressure; TG: triglycerides; HDL-C: high density lipoprotein cholesterol; CRP: C-reactive protein; Cr: serum creatinine; UA: uric acid; TC: serum total cholesterol; LDL-C: low density lipoprotein cholesterol; HbA1c: hemoglobin A1c; TNF-α: tumor necrosis factor-alpha.

**FIGURE 4:**
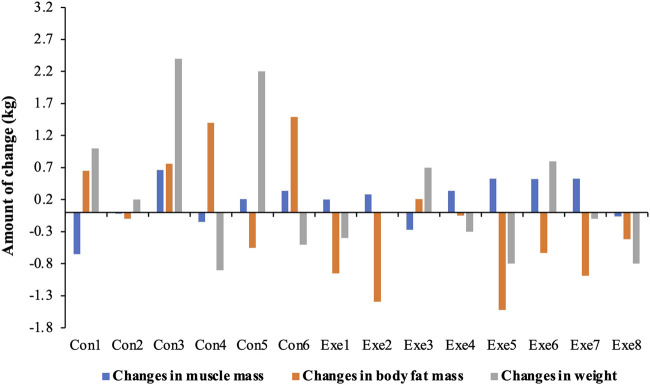
Changes in body composition after exercise training. Exe: combined aerobic and resistance exercise, Con: maintaining daily life.

Waist circumference, waist-hip ratio, BMI, skeletal muscle mass, and body fat mass are predictors of sarcopenic obesity. We found a significant group × time interaction for waist circumference (F = 6.254, *p* = 0.028) and waist-hip ratio (F = 5.935, *p* = 0.031). In the Con group, body fat mass increased significantly (F = 12.263, *p* = 0.004), which mainly reflected the waist circumference (F = 7.441, *p* = 0.018) and waist-hip ratio (F = 10.226, *p* = 0.008). The fat mass decreased significantly in the Exe group compared to that in the Con group (F = 8.392, *p* = 0.013). Moreover, skeletal muscle mass of subjects in the Exe group indicated a significant improvement after exercise intervention (*p* = 0.041) (Table 2, Table 3, and Figure 4).

#### 3.3.2 Changes in metabolic syndrome components between the study groups

The metabolic syndrome components were evaluated based on recommendations on metabolic syndrome from the Chinese Diabetes Society (Metabolic Syndrome Research Group of Chinese Medical Association, 2004), which included BMI, fasting blood glucose, blood pressure, triglycerides (TG), and high density lipoprotein cholesterol (HDL-C). Although a significant main effect of time was found for fasting blood glucose (F = 12.551, *p* = 0.004), only a significant reduction was observed in the Exe group. Furthermore, HDL-C showed a significant main effect of time (F = 46.862, *p* < 0.001). TG increased to a greater extent in the Con group compared to that in the Exe group (Table 3).

#### 3.3.3 Changes in other cardiovascular risk factors between the study groups

We also evaluated other risk factors for CVD, such as serum creatinine (Cr), serum total cholesterol (TC), cortisol (COR), C-reactive protein (CRP), hemoglobin A1c (HbA1c), and tumor necrosis factor-alpha (TNF-α). A significant group × time interaction was observed for COR (F = 7.084, *p* = 0.021), which showed a decrease in the Exe group and increase in the Con group after exercise intervention. In addition, significant main effect of time was detected for TC (F = 5.981, *p* = 0.031) and TNF-α (F = 36.730, *p* < 0.001). TC increased to a greater extent in the Con group compared to that in the Exe group (Tables 2, 3).

### 3.4 Relationship between changes in cardiovascular disease-related parameters and changes in relative abundance of gut microbiota after exercise intervention

**FIGURE 5:**
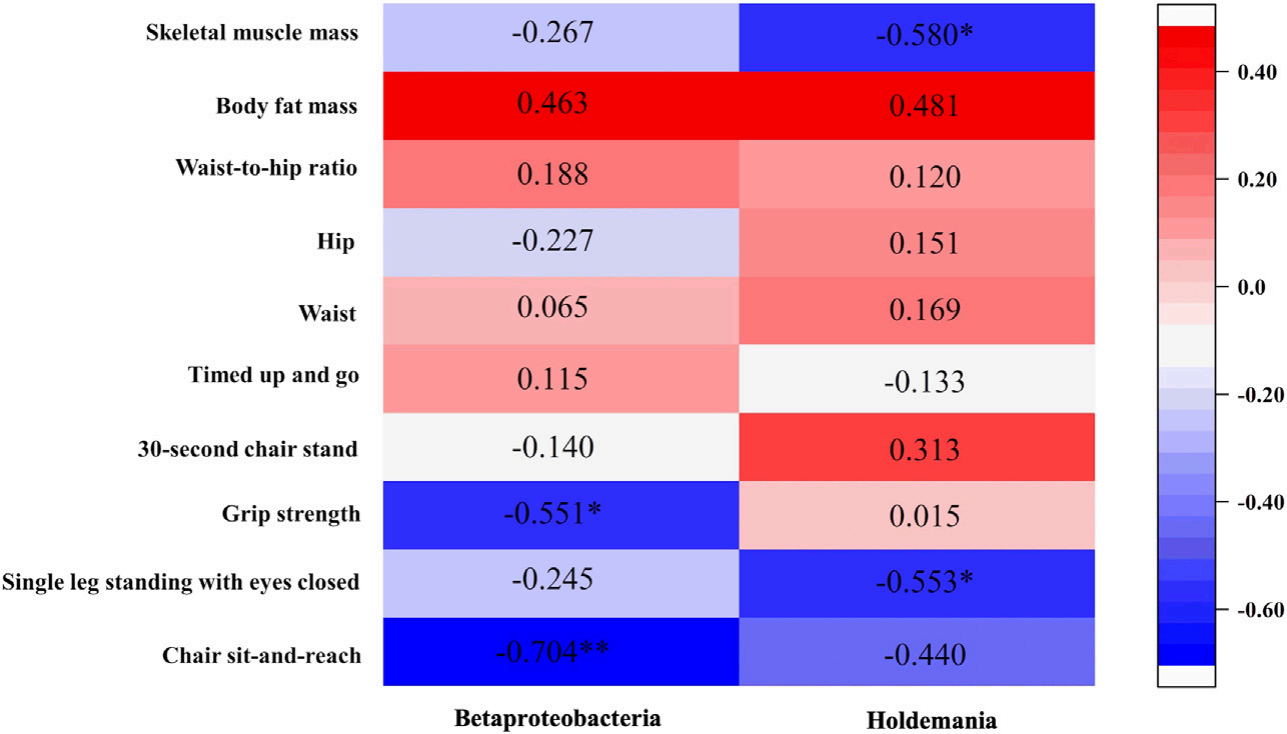
Relationship between changes in physical function and changes in gut microbiota after exercise intervention.

Chair sit-and-reach was negatively associated with Betaproteobacteria (*r* = −0.0704, *p* = 0.005). Single-leg standing with eyes closed was inversely correlated with *Holdemania* (*r* = −0.553, *p* = 0.040). Grip strength was negatively associated with Betaproteobacteria (*r* = −0.551, *p* = 0.041). Meanwhile, chair sit-and-reach was negatively associated with Betaproteobacteria (*r* = −0.704, *p* = 0.005). Skeletal muscle mass was negatively associated with *Holdemania* (*r* = −0.580, *p* = 0.030) (Figure 5).

## 4 Discussion

We believe this study is the first RCT to evaluate the effects of combined aerobic and resistance training on the gut microbiota and cardiovascular risk factors in physically active elderly women. Our main findings showed that the 8-week exercise program could improve physical function, lower CVD risk, alter alpha and beta diversity of the gut microbiota, and modulate the relative abundance of gut microbiota associated with CVD for the study participants. These findings indicate that exercise may still be effective in older adults that include exercise in their routines for preventing and controlling the risk of developing CVD.

Sarcopenic obesity is a strong predictor of CVD (Evans et al., 2021), characterized by the combination of low muscle mass (sarcopenia) and excess adiposity (obesity), which are evaluated according to skeletal muscle mass, fat mass, physical function, waist circumference, and BMI (Lee et al., 2016). Studies have shown that the skeletal muscle mass of elderly individuals who participated in combined aerobic and resistance exercise is increased and body fat is decreased (Chen et al., 2017), similar findings were also observed in our results. In addition to the improvement of body composition, we also observed enhancements in several physical function indicators such as balance function and flexibility, which were closely associated with the fall risk (Emilio et al., 2014). In fact, muscle weakness and poor balance ability are the most important risk factors of fall according to the ranking, and improvements in these functions can induce a reduction in fall risk (Rubenstein, 2006). In this study, exercise training ameliorates the muscle function and balance ability, which is consistent with previous study (Villareal et al., 2017). Thus, these findings suggest that the combination of structured aerobic and resistance training may reduce the risk of sarcopenic obesity and falls associated with impaired physical function in older adults.

A reduced risk of metabolic syndrome has also observed in exercise groups, which is important in that metabolic syndrome is a precursor of CVD (Wilson et al., 2005). A 12-week regimen of resistance band exercise training has been shown to improved glucose and blood lipid profile in obese postmenopausal women, thereby reducing the risk factors for metabolic syndrome (Son and Park, 2021). Both combined high-intensity aerobic and resistance exercise and low-intensity physical activity programs can improve some parameters of metabolic syndrome in elderly women, such as blood pressure, waist circumference, and TG (Kemmler et al., 2009). In our study, exercise significantly improved blood glucose of the participants, while TG in the Con group showed an increasing trend after 8 weeks. However, the HDL-C of individuals showed an overall decreasing trend in both study groups after intervention, which accordingly may have been related to temperature. The HDL-C is negatively associated with environmental temperature, being higher in the winter and lower in the spring (Wang et al., 2020). In our current study, the pre-test season was winter with the average temperature in the first 5 days of physical examination being 8 °C. The post-test season had just entered early spring with the average temperature in the first 5 days of physical examination being 9.9°C. The HDL-C of individuals showed an overall decreasing trend in both study groups after intervention, which accordingly may have been related to temperature.

In regard to other CVD risk factors, a previous study indicated a trend for greater increased total cholesterol of the control group compared to that of the exercise group after an 8-week combination of aerobic and resistance exercise (Ho et al., 2012). This finding is consistent with our current results. Moreover, our exercise training induced a significant decrease in the pro-inflammatory marker cortisol, which was similar to a previous study (Roberts et al., 2013). In addition, consideration of seasonal factors is important in evaluating inflammatory markers, including TNF-a, IL-6, IL-10, and CRP (Stewart et al., 2007; McFarlane et al., 2012). Serum creatinine of children with urinary 1-hydroxypyrene exhibits significant seasonal differences with it being higher in cold seasons compared to that in warm seasons (Chen et al., 2015). There were some seasonal differences before and after the intervention program of our study. Therefore, the significant consistent changes of serum creatinine and TNF-α in the Exe group and Con group may have been season associated.

Our findings also indicate that exercise could influence gut microbiota composition. Greater gut microbiota diversity is more protective for maintaining intestinal homeostasis, improving the integrity of the intestinal mucosal barrier (Human Microbiome Project, 2012; Faith et al., 2013), and resisting the harmful factors in the environment (Sommer et al., 2017). Conversely, a lack of gut microbiota diversity in humans is associated with multiple diseases, including type 2 diabetes (Miao et al., 2020) and CVD (Kelly et al., 2016). Thus, a more diverse gut microbiota can be considered beneficial to health. Previous studies indicate that exercise training can contribute to higher alpha diversity (Hampton-Marcell et al., 2020), which was athletes who participated in high-intensity and great-frequency training. Patients with quiescent inflammatory bowel disease undergoing a moderate-intensity progressive combination of aerobic and resistance exercise (3 times per week for 8 weeks) also show a slight increase in alpha diversity after the exercise intervention (Cronin et al., 2019). However, there are also several previous reports suggesting that moderate and low-to-moderate intensity aerobic exercise (or combined aerobic and resistance exercise) performed three times a week for durations ranging from 5 to 8 weeks did not significantly affect the gut microbiota alpha diversity of elderly individuals (Taniguchi et al., 2018), overweight or obese people (Cronin et al., 2018), or sedentary people (Munukka et al., 2018). Of note, our results show that an 8-week exercise training four times per week could significantly increase some indicators of alpha diversity of gut microbiota in active elderly people. Therefore, increasing frequency of exercise during short-term exercise interventions may partially increase the alpha diversity of gut microbiota.

The association between parameters of sarcopenic obesity and gut microbiota have been only rarely reported. Our results revealed a possible relationship between these parameters and consistent with previous studies. For example, flexibility (chair sit-and-reach) and muscle strength (grip strength-weight ratio) were inversely correlated with Betaproteobacteria, a class of bacteria associated with obesity (Quiroga et al., 2020). Skeletal muscle mass was negatively associated with *Holdemania*, which is associated with impaired glucose metabolism (Lippert et al., 2017). Previous studies have shown that Betaproteobacteria is more abundant in obese individuals compared to that in healthy individuals (Quiroga et al., 2020). Our current study indicated that prevalence of Betaproteobacteria decreased slightly after exercise intervention and the risk of obesity decreased for the Exe group. These changes showed opposite trends in the Con group. A higher abundance of *Fusicatenibacter* is observed in non-obese adults compared to that in obese adults (Jonduo et al., 2020) and exercise training significantly increased the relative abundance of this bacteria in our study. Still, other bacteria associated with obesity, such as the genus *Coprococcus*, are higher in non-obese subjects compare to that in obese subjects (Jonduo et al., 2020). It is reported that exercise training can increase the relative abundance of *Coprococcus* (Hampton-Marcell et al., 2020), which is consistent with our current results. Moreover, there is increasing evidence that suggests *Sutterella* is more abundant in obese individuals compared to that in individuals of normal weight (Chen et al., 2020). Our results indicated an increase of *Sutterella* in the Con group. Thus, according to our results and previous findings, these bacteria may be associated with sarcopenic obesity.

With respect to the association between gut microbiota and glycolipid metabolism, a previous study revealed the relative abundance of the genus *Holdemania* is associated with impaired lipid and glucose metabolism in older adults (Lippert et al., 2017). Although our study did not observe a statistically significant association between *Holdemania* and glycolipid metabolism, the abundance of this bacteria was decreased in the Exe group after exercise intervention. Notably, the family Coriobacteriaceae is considered to be a contributor to some metabolic functions, such as glucose homeostasis and lipid metabolism (Clavel et al., 2014; Liu et al., 2018) and higher abundance of Coriobacteriaceae members may be beneficial to the host (Kim et al., 2020). Moreover, a higher proportion of *Collinsella* has also been observed in overweight or obese women with better lipid profiles (Gomes et al., 2019). Our current results indicated a significant increase for the two bacteria in the Exe group after intervention. Thus, exercise may modulate the abundance of these bacteria and thereby reduce the risk of CVD.

The potential mechanisms of exercise affecting cardiovascular and metabolic risk factors may be mediated through microbial metabolic pathways. Exercise is known to induce increased concentrations of short-chain fatty acids (SCFAs) such as butyrate, which are metabolized by gut microbiota (Allen et al., 2018). Many bacteria have the ability to produce SCFAs, including Clostridia (Louis and Flint, 2009) and Ruminococcaceae (Choi et al., 2021). These gut microbiota can regulate blood pressure, body fat, and glucose by producing SCFAs (Morrison and Preston, 2016), which showed an increasing trend in our study after exercise intervention compared to that of the Con group. Furthermore, it has been previously reported that SCFAs can induce a decrease in blood pressure (Onyszkiewicz et al., 2019) and fat mass (Sahuri-Arisoylu et al., 2016). Host SCFA G-protein-coupled receptors may play a role in SCFA-mediated host-microbe communication in regulating blood pressure (Poll et al., 2020) and fat mass development (Lu et al., 2016). In addition, intestinal permeability, low-grade endotoxemia, and branched-chain amino acids may all be involved in possible mechanisms linking the gut microbiota and glucose homeostasis (Utzschneider et al., 2016). Meanwhile, regular exercise can protect the function and permeability of the gut barrier and promote the gut microbiota in supporting a healthy phenotype (Fiuza-Luces et al., 2018). Also, crosstalk between the gut microbiota and skeletal muscle is a two-way process and changes in the gut microbiota may modulate skeletal muscle bioenergetics by altering substrate availability (Hawley, 2020). However, the precise mechanism of the interaction between exercise, the gut microbiota, and CVD risk remains to be elucidated.

Several limitations of our current study should be considered. First, confounding factors were not considered when observing the effects of exercise training on gut microbiota composition and cardiovascular disease risk, which may partially influence our findings. In order to lower the influence of confounding factors such as dietary intakes (Scott et al., 2013), the RCT was designed, which can optimize the likelihood that confounding variables from measurement and non-measurement are distributed equally, and enable any difference in results to be attributed to the exercise training (Handley et al., 2018). Second, the absence analysis of gut microbiota metabolites may influence our further discussion regarding the mechanisms of exercise affecting CVD risk. Third, some results and findings from this study may be underestimated due to the relatively small sample size and further studies using large sample sizes are needed.

## 5 Conclusion

Our study indicated that an 8-week regimen of combined aerobic and resistance training could improve the physical function of physically active elderly women and lower their risk of sarcopenic obesity and metabolic syndrome. Exercise altered the alpha diversity of the gut microbiota and modulated the relative abundance of gut microbiota associated with CVD, such as Betaproteobacteria and *Holdemania*. In the future, metabolic analyses will be necessary to further interpret these findings and identify the potential pathways involved.

## Data Availability

Raw reads were uploaded to the NCBI SRA (National Center for Biotechnology information Sequence Read Archive) database under BioProject ID PRJNA763801 (http://www.ncbi.nlm.nih.gov/bioproject/763801). The other datasets supporting these studies conclusion are available from the corresponding author.

## References

[B1] Allen J. M. Mailing L. J. Niemiro G. M. Moore R. Cook M. D. While B. A. (2018). Exercise alters gut microbiota composition and function in lean and obese humans. Med. Sci. Sports Exerc. 50 (4), 747–757. 10.1249/MSS.0000000000001495 29166320

[B2] Beavers K. M. Walkup M. P. Weaver A. A. Lenchik L. Kritchevsky S. B. Nicklas B. J. (2018). Effect of exercise modality during weight loss on bone health in older adults with obesity and cardiovascular disease or metabolic syndrome: A randomized controlled trial. J. Bone Min. Res. 33 (12), 2140–2149. 10.1002/jbmr.3555 PMC6545884 30088288

[B3] Bermon S. Petriz B. Kajeniene A. Prestes J. Castell L. Franco O. L. (2015). The microbiota: An exercise immunology perspective. Exerc. Immunol. Rev. 21, 70–79. 25825908

[B4] Cannon B. (2013). Cardiovascular disease: Biochemistry to behaviour. Nature 493 (7434), S2–S3. 10.1038/493S2a 23364768

[B67] Caporaso J. G. Kuczynski J. Stombaugh J. Bittinger K. Bushman F. D. Costello E. K. (2010). QIIME allows analysis of high-throughput community sequencing data. Nat. Methods 7 (5), 335–336. 10.1038/nmeth.f.303 20383131 PMC3156573

[B5] Chen H. T. Chung Y. C. Chen Y. J. Ho S. Y. Wu H. J. (2017). Effects of different types of exercise on body composition, muscle strength, and IGF-1 in the elderly with sarcopenic obesity. J. Am. Geriatr. Soc. 65 (4), 827–832. 10.1111/jgs.14722 28205203

[B6] Chen X. Sun H. Jiang F. Shen Y. Li X. Hu X. (2020). Alteration of the gut microbiota associated with childhood obesity by 16S rRNA gene sequencing. PeerJ 8, e8317. 10.7717/peerj.8317 31976177 PMC6968493

[B7] Chen Y. T. Huang Y. K. Luvsan M. E. Gombojav E. Ochir C. Bulgan J. (2015). The influence of season and living environment on children's urinary 1-hydroxypyrene levels in Ulaanbaatar, Mongolia. Environ. Res. 137, 170–175. 10.1016/j.envres.2014.11.022 25543547

[B8] Choi S. I. Son J. H. Kim N. Kim Y. S. Nam R. H. Park J. H. (2021). Changes in cecal microbiota and short-chain fatty acid during lifespan of the rat. J. Neurogastroenterol. Motil. 27 (1), 134–146. 10.5056/jnm20148 33380558 PMC7786083

[B9] Clavel T. Desmarchelier C. Haller D. Gerard P. Rohn S. Lepage P. (2014). Intestinal microbiota in metabolic diseases: From bacterial community structure and functions to species of pathophysiological relevance. Gut Microbes 5 (4), 544–551. 10.4161/gmic.29331 25003516

[B10] Cronin O. Barton W. Moran C. Sheehan D. Whiston R. Nugent H. (2019). Moderate-intensity aerobic and resistance exercise is safe and favorably influences body composition in patients with quiescent inflammatory bowel disease: A randomized controlled cross-over trial. BMC Gastroenterol. 19 (1), 29. 10.1186/s12876-019-0952-x 30755154 PMC6373036

[B11] Cronin O. Barton W. Skuse P. Penney N. C. Garcia-Perez I. Murphy E. F. (2018). A prospective metagenomic and metabolomic analysis of the impact of exercise and/or whey protein supplementation on the gut microbiome of sedentary adults. mSystems 3 (3), e00044-18. 10.1128/mSystems.00044-18 29719871 PMC5915698

[B12] Curcio F. Liguori I. Cellulare M. Sasso G. Della-Morte D. Gargiulo G. (2019). Physical activity Scale for the elderly (PASE) score is related to sarcopenia in noninstitutionalized older adults. J. Geriatr. Phys. Ther. 42 (3), 130–135. 10.1519/JPT.0000000000000139 28786911

[B68] Edgar R. C. (2010). Search and clustering orders of magnitude faster than BLAST. Bioinformatics 26 (19), 2460–2461. 10.1093/bioinformatics/btq461 20709691

[B69] Edgar R. C. (2013). UPARSE: highly accurate OTU sequences from microbial amplicon reads. Nat. Methods 10 (10), 996–998. 10.1038/nmeth.2604 23955772

[B70] Edgar R. C. Haas B. J. Clemente J. C. Quince C. Knight R. (2011). UCHIME improves sensitivity and speed of chimera detection. Bioinformatics 27 (16), 2194–2200. 10.1093/bioinformatics/btr381 21700674 PMC3150044

[B13] Emilio E. J. Hita-Contreras F. Jimenez-Lara P. M. Latorre-Roman P. Martinez-Amat A. (2014). The association of flexibility, balance, and lumbar strength with balance ability: Risk of falls in older adults. J. Sports Sci. Med. 13 (2), 349–357. 24790489 PMC3990889

[B14] Evans K. Abdelhafiz D. Abdelhafiz A. H. (2021). Sarcopenic obesity as a determinant of cardiovascular disease risk in older people: A systematic review. Postgrad. Med. 133 (8), 831–842. 10.1080/00325481.2021.1942934 34126036

[B15] Faith J. J. Guruge J. L. Charbonneau M. Subramanian S. Seedorf H. Goodman A. L. (2013). The long-term stability of the human gut microbiota. Science 341 (6141), 1237439. 10.1126/science.1237439 23828941 PMC3791589

[B16] Fiuza-Luces C. Santos-Lozano A. Joyner M. Carrera-Bastos P. Picazo O. Zugaza J. L. (2018). Exercise benefits in cardiovascular disease: Beyond attenuation of traditional risk factors. Nat. Rev. Cardiol. 15 (12), 731–743. 10.1038/s41569-018-0065-1 30115967

[B17] Gao F. Gao E. Yue T. L. Ohlstein E. H. Lopez B. L. Christopher T. A. (2002). Nitric oxide mediates the antiapoptotic effect of insulin in myocardial ischemia-reperfusion: The roles of PI3-kinase, akt, and endothelial nitric oxide synthase phosphorylation. Circulation 105 (12), 1497–1502. 10.1161/01.cir.0000012529.00367.0f 11914261

[B18] Gomes A. C. Hoffmann C. Mota J. F. (2019). Gut microbiota is associated with adiposity markers and probiotics may impact specific genera. Eur. J. Nutr. 59 (4), 1751–1762. 10.1007/s00394-019-02034-0 31250099

[B19] Hampton-Marcell J. T. Eshoo T. W. Cook M. D. Gilbert J. A. Horswill C. A. Poretsky R. (2020). Comparative analysis of gut microbiota following changes in training volume Among swimmers. Int. J. Sports Med. 41 (5), 292–299. 10.1055/a-1079-5450 31975357

[B20] Handley M. A. Lyles C. R. McCulloch C. Cattamanchi A. (2018). Selecting and improving quasi-experimental designs in effectiveness and implementation research. Annu. Rev. Public Health 39, 5–25. 10.1146/annurev-publhealth-040617-014128 29328873 PMC8011057

[B21] Hawley J. A. (2020). Microbiota and muscle highway - two way traffic. Nat. Rev. Endocrinol. 16 (2), 71–72. 10.1038/s41574-019-0291-6 31728050

[B22] Ho S. S. Dhaliwal S. S. Hills A. P. Pal S. (2012). The effect of 12 weeks of aerobic, resistance or combination exercise training on cardiovascular risk factors in the overweight and obese in a randomized trial. BMC Public Health 12, 704. 10.1186/1471-2458-12-704 23006411 PMC3487794

[B23] Holland A. M. Hyatt H. W. Smuder A. J. Sollanek K. J. Morton A. B. Roberts M. D. (2015). Influence of endurance exercise training on antioxidant enzymes, tight junction proteins, and inflammatory markers in the rat ileum. BMC Res. Notes 8, 514. 10.1186/s13104-015-1500-6 26423686 PMC4589170

[B24] Human Microbiome Project C. (2012). Structure, function and diversity of the healthy human microbiome. Nature 486 (7402), 207–214. 10.1038/nature11234 22699609 PMC3564958

[B25] Jonduo M. E. Wawae L. Masiria G. Suda W. Hattori M. Takayasu L. (2020). Gut microbiota composition in obese and non-obese adult relatives from the highlands of Papua New Guinea. FEMS Microbiol. Lett. 367 (19), fnaa161. 10.1093/femsle/fnaa161 33021675

[B26] Kelly T. N. Bazzano L. A. Ajami N. J. He H. Zhao J. Petrosino J. F. (2016). Gut microbiome associates with lifetime cardiovascular disease risk profile Among bogalusa heart study participants. Circ. Res. 119 (8), 956–964. 10.1161/CIRCRESAHA.116.309219 27507222 PMC5045790

[B27] Kemmler W. Von Stengel S. Engelke K. Kalender W. A. (2009). Exercise decreases the risk of metabolic syndrome in elderly females. Med. Sci. Sports Exerc. 41 (2), 297–305. 10.1249/MSS.0b013e31818844b7 19127197

[B28] Kim M. H. Yun K. E. Kim J. Park E. Chang Y. Ryu S. (2020). Gut microbiota and metabolic health among overweight and obese individuals. Sci. Rep. 10 (1), 19417. 10.1038/s41598-020-76474-8 33173145 PMC7655835

[B29] Kyu H. H. Abate D. Abate K. H. Abay S. M. Abbafati C. Abbasi N. (2018). Global, regional, and national disability-adjusted life-years (DALYs) for 359 diseases and injuries and healthy life expectancy (HALE) for 195 countries and territories, 1990-2017: A systematic analysis for the global burden of disease study 2017. Lancet 392 (10159), 1859–1922. 10.1016/S0140-6736(18)32335-3 30415748 PMC6252083

[B30] Lakka H. M. Laaksonen D. E. Lakka T. A. Niskanen L. K. Kumpusalo E. Tuomilehto J. (2002). The metabolic syndrome and total and cardiovascular disease mortality in middle-aged men. JAMA 288 (21), 2709–2716. 10.1001/jama.288.21.2709 12460094

[B31] Lee D. C. Shook R. P. Drenowatz C. Blair S. N. (2016). Physical activity and sarcopenic obesity: Definition, assessment, prevalence and mechanism. Future Sci. OA 2 (3), FSO127. 10.4155/fsoa-2016-0028 28031974 PMC5137918

[B32] Li H. Qin S. Liang Q. Xi Y. Bo W. Cai M. (2021). Exercise training enhances myocardial mitophagy and improves cardiac function via irisin/FNDC5-PINK1/parkin pathway in MI mice. Biomedicines 9 (6), 701. 10.3390/biomedicines9060701 34205641 PMC8234442

[B33] Lippert K. Kedenko L. Antonielli L. Kedenko I. Gemeier C. Leitner M. (2017). Gut microbiota dysbiosis associated with glucose metabolism disorders and the metabolic syndrome in older adults. Benef. Microbes 8 (4), 545–556. 10.3920/BM2016.0184 28701081

[B34] Liu H. Zhang H. Wang X. Yu X. Hu C. Zhang X. (2018). The family Coriobacteriaceae is a potential contributor to the beneficial effects of Roux-en-Y gastric bypass on type 2 diabetes. Surg. Obes. Relat. Dis. 14 (5), 584–593. 10.1016/j.soard.2018.01.012 29459013

[B35] Louis P. Flint H. J. (2009). Diversity, metabolism and microbial ecology of butyrate-producing bacteria from the human large intestine. FEMS Microbiol. Lett. 294 (1), 1–8. 10.1111/j.1574-6968.2009.01514.x 19222573

[B36] Lu Y. Fan C. Li P. Lu Y. Chang X. Qi K. (2016). Short chain fatty acids prevent high-fat-diet-induced obesity in mice by regulating G protein-coupled receptors and gut microbiota. Sci. Rep. 6, 37589. 10.1038/srep37589 27892486 PMC5124860

[B71] Magoc T. Salzberg S. L. (2011). FLASH: fast length adjustment of short reads to improve genome assemblies. Bioinformatics 27 (21), 2957–2963. 10.1093/bioinformatics/btr507 21903629 PMC3198573

[B37] Mailing L. J. Allen J. M. Buford T. W. Fields C. J. Woods J. A. (2019). Exercise and the gut microbiome: A review of the evidence, potential mechanisms, and implications for human health. Exerc. Sport Sci. Rev. 47 (2), 75–85. 10.1249/JES.0000000000000183 30883471

[B38] Matsubara Y. Matsumoto T. Inoue K. Matsuda D. Yoshiga R. Yoshiya K. (2017). Sarcopenia is a risk factor for cardiovascular events experienced by patients with critical limb ischemia. J. Vasc. Surg. 65 (5), 1390–1397. 10.1016/j.jvs.2016.09.030 27986478

[B39] McFarlane D. Wolf R. F. McDaniel K. A. White G. L. (2012). The effect of season on inflammatory response in captive baboons. J. Med. Primatol. 41, 341–348. 10.1111/j.1600-0684.2012.00560.x 22905903 PMC3492523

[B40] Metabolic Syndrome Research Group of Chinese Medical Association (2004). Suggestions on metabolic syndrome from Chinese diabetes society. Chin. J. Diabetes 12 (3), 156–161. 10.3321/j.issn:1006-6187.2004.03.002

[B41] Miao Z. Lin J. S. Mao Y. Chen G. D. Zeng F. F. Dong H. L. (2020). Erythrocyte n-6 polyunsaturated fatty acids, gut microbiota, and incident type 2 diabetes: A prospective cohort study. Diabetes Care 43 (10), 2435–2443. 10.2337/dc20-0631 32723842 PMC7510039

[B42] Morrison D. J. Preston T. (2016). Formation of short chain fatty acids by the gut microbiota and their impact on human metabolism. Gut Microbes 7 (3), 189–200. 10.1080/19490976.2015.1134082 26963409 PMC4939913

[B43] Munukka E. Ahtiainen J. P. Puigbó P. Jalkanen S. Pahkala K. Keskitalo A. (2018). Six-week endurance exercise alters gut metagenome that is not reflected in systemic metabolism in over-weight women. Front. Microbiol. 9, 2323. 10.3389/fmicb.2018.02323 30337914 PMC6178902

[B72] Ohara M. Kohara K. Tabara Y. Igase M. Miki T. (2015). Portable indices for sarcopenia are associated with pressure wave reflection and central pulse pressure: the J-SHIPP study. J. Hypertens 33 (2), 314–322. 10.1097/HJH.0000000000000394 25380165

[B73] Ohara M. Kohara K. Tabara Y. Ochi M. Nagai T. Igase M. (2014). Sarcopenic obesity and arterial stiffness, pressure wave reflection and central pulse pressure: the J-SHIPP study. Int. J. Cardiol. 174 (1), 214–217. 10.1016/j.ijcard.2014.03.194 24767131

[B44] Onyszkiewicz M. Gawrys-Kopczynska M. Konopelski P. Aleksandrowicz M. Sawicka A. Kozniewska E. (2019). Butyric acid, a gut bacteria metabolite, lowers arterial blood pressure via colon-vagus nerve signaling and GPR41/43 receptors. Pflugers Arch. 471 (11-12), 1441–1453. 10.1007/s00424-019-02322-y 31728701 PMC6882756

[B74] Podsiadlo D. Richardson S. (1991). The timed “Up & Go”: a test of basic functional mobility for frail elderly persons. J. Am. Geriatr. Soc. 39 (2), 142–148. 10.1111/j.1532-5415.1991.tb01616.x 1991946

[B45] Poll B. G. Cheema M. U. Pluznick J. L. (2020). Gut microbial metabolites and blood pressure regulation: Focus on SCFAs and TMAO. Physiol. (Bethesda) 35 (4), 275–284. 10.1152/physiol.00004.2020 PMC7474256 32490748

[B46] Quiroga R. Nistal E. Estébanez B. Porras D. Juárez-Fernández M. Martínez-Flórez S. (2020). Exercise training modulates the gut microbiota profile and impairs inflammatory signaling pathways in obese children. Exp. Mol. Med. 52 (7), 1048–1061. 10.1038/s12276-020-0459-0 32624568 PMC8080668

[B47] Ramakrishnan R. Doherty A. Smith-Byrne K. Rahimi K. Bennett D. Woodward M. (2021). Accelerometer measured physical activity and the incidence of cardiovascular disease: Evidence from the UK Biobank cohort study. PLoS Med. 18 (1), e1003487. 10.1371/journal.pmed.1003487 33434193 PMC7802951

[B75] Rikli R. E. Jones C. J. (1999). Development and validation of a functional fitness test for community-residing older adults. J. Aging Phys. Act. 7 (2), 129–161. 10.1123/japa.7.2.129

[B48] Roberts C. K. Croymans D. M. Aziz N. Butch A. W. Lee C. C. (2013). Resistance training increases SHBG in overweight/obese, young men. Metabolism. 62 (5), 725–733. 10.1016/j.metabol.2012.12.004 23318050 PMC3845495

[B49] Rubenstein L. Z. (2006). Falls in older people: Epidemiology, risk factors and strategies for prevention. Age Ageing 35, ii37–ii41. 10.1093/ageing/afl084 16926202

[B50] Sahuri-Arisoylu M. Brody L. P. Parkinson J. R. Parkes H. Navaratnam N. Miller A. D. (2016). Reprogramming of hepatic fat accumulation and 'browning' of adipose tissue by the short-chain fatty acid acetate. Int. J. Obes. 40 (6), 955–963. 10.1038/ijo.2016.23 26975441

[B51] Schroeder E. C. Franke W. D. Sharp R. L. Lee D. C. (2019). Comparative effectiveness of aerobic, resistance, and combined training on cardiovascular disease risk factors: A randomized controlled trial. PLoS One 14 (1), e0210292. 10.1371/journal.pone.0210292 30615666 PMC6322789

[B52] Scott K. P. Gratz S. W. Sheridan P. O. Flint H. J. Duncan S. H. (2013). The influence of diet on the gut microbiota. Pharmacol. Res. 69 (1), 52–60. 10.1016/j.phrs.2012.10.020 23147033

[B53] Sommer F. Anderson J. M. Bharti R. Raes J. Rosenstiel P. (2017). The resilience of the intestinal microbiota influences health and disease. Nat. Rev. Microbiol. 15 (10), 630–638. 10.1038/nrmicro.2017.58 28626231

[B54] Son W.-M. Park J.-J. (2021). Resistance band exercise training prevents the progression of metabolic syndrome in obese postmenopausal women. J. Sports Sci. Med. 20, 291–299. 10.52082/jssm.2021.291 34211322 PMC8219266

[B55] Song B. K. Cho K. O. Jo Y. Oh J. W. Kim Y. S. (2012). Colon transit time according to physical activity level in adults. J. Neurogastroenterol. Motil. 18 (1), 64–69. 10.5056/jnm.2012.18.1.64 22323989 PMC3271256

[B56] Stewart N. Taylor B. Ponsonby A. L. Pittas F. van der Mei I. Woods G. (2007). The effect of season on cytokine expression in multiple sclerosis and healthy subjects. J. Neuroimmunol. 188 (1-2), 181–186. 10.1016/j.jneuroim.2007.06.012 17628701

[B57] Taniguchi H. Tanisawa K. Sun X. Kubo T. Hoshino Y. Hosokawa M. (2018). Effects of short-term endurance exercise on gut microbiota in elderly men. Physiol. Rep. 6 (23), e13935. 10.14814/phy2.13935 30536648 PMC6286434

[B58] Thompson P. D. Buchner D. Pina I. L. Balady G. J. Williams M. A. Marcus B. H. (2003). Exercise and physical activity in the prevention and treatment of atherosclerotic cardiovascular disease: A statement from the council on clinical cardiology (subcommittee on exercise, rehabilitation, and prevention) and the council on nutrition, physical activity, and metabolism (subcommittee on physical activity). Circulation 107 (24), 3109–3116. 10.1161/01.CIR.0000075572.40158.77 12821592

[B59] Utzschneider K. M. Kratz M. Damman C. J. Hullar M. (2016). Mechanisms linking the gut microbiome and glucose metabolism. J. Clin. Endocrinol. Metab. 101 (4), 1445–1454. 10.1210/jc.2015-4251 26938201 PMC4880177

[B60] Villareal D. T. Aguirre L. Gurney A. B. Waters D. L. Sinacore D. R. Colombo E. (2017). Aerobic or resistance exercise, or both, in dieting obese older adults. N. Engl. J. Med. 376 (20), 1943–1955. 10.1056/NEJMoa1616338 28514618 PMC5552187

[B61] Wang D. Yu S. Zou Y. Li H. Cheng X. Qiu L. (2020). Data mining: Seasonal fluctuations and associations between thyroid stimulating hormone and lipid profiles. Clin. Chim. Acta. 506, 122–128. 10.1016/j.cca.2020.03.012 32165124

[B62] Wang Y. Liu J. Wang W. Wang M. Qi Y. Xie W. (2015). Lifetime risk for cardiovascular disease in a Chinese population: The Chinese multi-provincial cohort study. Eur. J. Prev. Cardiol. 22 (3), 380–388. 10.1177/2047487313516563 24336461

[B63] Wilson P. W. D'Agostino R. B. Parise H. Sullivan L. Meigs J. B. (2005). Metabolic syndrome as a precursor of cardiovascular disease and type 2 diabetes mellitus. Circulation 112 (20), 3066–3072. 10.1161/CIRCULATIONAHA.105.539528 16275870

[B64] Witkowski M. Weeks T. L. Hazen S. L. (2020). Gut microbiota and cardiovascular disease. Circ. Res. 127 (4), 553–570. 10.1161/CIRCRESAHA.120.316242 32762536 PMC7416843

[B65] Yusuf S. Joseph P. Rangarajan S. Islam S. Mente A. Hystad P. (2020). Modifiable risk factors, cardiovascular disease, and mortality in 155 722 individuals from 21 high-income, middle-income, and low-income countries (PURE): A prospective cohort study. Lancet 395 (10226), 795–808. 10.1016/S0140-6736(19)32008-2 31492503 PMC8006904

[B66] Zhong F. Wen X. Yang M. Lai H. Y. Momma H. Cheng L. (2020). Effect of an 8-week exercise training on gut microbiota in physically inactive older women. Int. J. Sports Med. 42, 610–623. 10.1055/a-1301-7011 33321523

