# Supplementary material for: Effects of combined aerobic and resistance training on gut microbiota and cardiovascular risk factors in physically active elderly women: A randomized controlled trial
Source: Front Physiol. 2022 Oct 20;13:1004863. doi: 10.3389/fphys.2022.1004863 (PMC9631483; doi:10.3389/fphys.2022.1004863)
Supplement: Supplementary file 1 [file Table1.docx]

Table S1. Changes in alpha diversity, phylum, class, order levels of gut microbiota in each group following the intervention.

| Taxa | speciﬁc | Exercise group (n=8) | | | Control group (n=6) | | | Main effect (time) | | | Main effect (group) | | | Interaction  (Group × Time) | |
| --- | --- | --- | --- | --- | --- | --- | --- | --- | --- | --- | --- | --- | --- | --- | --- |
|  |  | Baseline | After intervention | P | Baseline | After intervention | P | F | P | Mean difference  (95% CI) | F | P | Mean difference  (95% CI) | F | P |
| Alpha diversity | Sobs (OTU) | 137.13 ± 49.65 | 168.13 ± 52.13 | 0.008 | 114.67 ± 39.47 | 147.00 ± 36.61 | 0.086 | 15.273 | 0.002 | 31.667 (14.012, 49.321) | 0.861 | 0.372 | 21.792 (-29.368, 72.951) | 0.007 | 0.936 |
|  | Chao | 155.29 ± 52.03 | 184.69 ± 53.70 | 0.017 | 129.04 ± 37.33 | 165.31 ± 41.97 | 0.097 | 13.402 | 0.003 | 32.833 (13.292, 52.375) | 0.887 | 0.365 | 22.808 (-29.971, 75.587) | 0.147 | 0.708 |
|  | Ace | 149.02 ± 51.51 | 184.71 ± 53.51 | 0.002 | 131.92 ± 38.43 | 168.18 ± 43.27 | 0.098 | 16.637 | 0.002 | 35.973 (16.757, 55.189) | 0.475 | 0.504 | 16.815 (-36.334, 69.965) | 0.001 | 0.974 |
|  | Shannon | 2.54 ± 0.86 | 2.89 ± 0.70 | 0.177 | 2.55 ± 0.75 | 2.80 ± 0.52 | 0.376 | 2.950 | 0.112 | 0.302 (-0.081, 0.685) | 0.010 | 0.923 | 0.035 (-0.735, 0.805) | 0.069 | 0.798 |
|  | Simpson | 0.207 ± 0.195 | 0.142 ± 0.110 | 0.401 | 0.190 ± 0.131 | 0.151 ± 0.098 | 0.486 | 1.500 | 0.244 | -0.052 (-0.146, 0.041) | 0.005 | 0.948 | 0.004 (-0.135, 0.143) | 0.093 | 0.766 |
| Phylum | Actinobacteria | 2.78 ± 5.78 | 3.21 ± 6.34 | 0.484 | 1.00 ± 1.20 | 2.82 ± 3.78 | 0.249 | 0.363 | 0.558 | 1.125 (-2.945, 5.195) | 0.319 | 0.583 | 1.091 (-3.117, 5.299) | 0.138 | 0.716 |
|  | Bacteroidetes | 35.63 ± 23.74 | 35.15 ± 28.55 | 0.954 | 53.59 ± 7.55 | 55.14 ± 11.95 | 0.768 | 0.011 | 0.917 | 0.538 (-10.543, 11.619) | 3.476 | 0.087 | -18.975 (-41.149, 3.199) | 0.040 | 0.845 |
|  | Firmicutes | 46.81 ± 17.77 | 57.89 ± 26.90 | 0.123 | 40.75 ± 9.07 | 37.41 ± 13.61 | 0.463 | 0.383 | 0.548 | 3.868 (-9.756, 17.492) | 2.684 | 0.127 | 13.268 (-4.377, 30.913) | 1.331 | 0.271 |
|  | Proteobacteria | 11.83 ± 20.08 | 2.88 ± 3.33 | 0.208 | 3.88 ± 3.14 | 2.30 ± 1.36 | 0.917 | 1.524 | 0.241 | -5.265 (-14.556, 4.026) | 1.023 | 0.332 | 4.265 (-4.923, 13.452) | 0.747 | 0.404 |
| Class | Bacilli | 0.53 ± 0.46 | 0.20 ± 0.22 | 0.208 | 0.07 ± 0.05 | 0.130 ± 0.08 | 0.125 | 1.620 | 0.227 | -0.137 (-0.371, 0.097) | 6.336 | 0.027 | 0.261 (0.035, 0.487) | 3.318 | 0.094 |
|  | Clostridia | 27.12 ± 14.62 | 44.98 ± 24.05 | 0.095 | 28.09 ± 13.59 | 32.29 ± 14.16 | 0.290 | 3.875 | 0.073 | 11.032 (-1.179, 23.242) | 0.579 | 0.461 | 5.863 (-10.925, 22.650) | 1.483 | 0.247 |
|  | Erysipelotrichia | 0.107 ± 0.083 | 0.157 ± 0.072 | 0.242 | 0.469 ± 0.825 | 0.286 ± 0.309 | 0.917 | 0.269 | 0.614 | -0.067 (-0.348, 0.214) | 1.913 | 0.192 | -0.246 (-0.632, 0.141) | 0.820 | 0.383 |
|  | Fusobacteriia | 0.065 ± 0.144 | 0.030 ± 0.059 | 0.866 | 0.167 ± 0.326 | 0.715 ± 1.432 | 0.285 | 1.748 | 0.211 | 0.256 (-0.166, 0.678) | 1.620 | 0.227 | -0.394 (-1.068, 0.280) | 2.265 | 0.158 |
| Order | Coriobacteriales | 0.56 ± 1.11 | 2.75 ± 6.28 | 0.012 | 0.18 ± 0.29 | 0.35 ± 0.32 | 0.173 | 1.210 | 0.293 | 1.178 (-1.155, 3.512) | 0.836 | 0.379 | 1.394 (-1.929, 4.718) | 0.897 | 0.362 |
|  | Lactobacillales | 0.518 ± 0.442 | 0.197 ± 0.220 | 0.208 | 0.073 ± 0.044 | 0.130 ± 0.083 | 0.125 | 1.567 | 0.235 | -0.132 (-0.361, 0.097) | 6.378 | 0.027 | 0.256 (0.035, 0.477) | 3.234 | 0.097 |
| Family | Bacteroidaceae | 25.82 ± 22.56 | 19.11 ± 20.23 | 0.152 | 50.04 ± 10.21 | 51.80 ± 12.54 | 0.744 | 0.578 | 0.462 | -2.480 (-9.585, 4.626) | 9.711 | 0.009 | -28.451 (-48.344, -8.559) | 1.686 | 0.219 |
|  | Carnobacteriaceae | 0.005 ± 0.006 | 0.002 ± 0.003 | 0.345 | 0.001 ± 0.002 | 0.004 ± 0.005 | 0.273 | 0.009 | 0.925 | 0.000 (-0.003, 0.004) | 0.423 | 0.528 | 0.001 (-0.003, 0.005) | 2.193 | 0.164 |
|  | Coriobacteriaceae | 0.56 ± 1.11 | 2.75 ± 6.58 | 0.012 | 0.18 ± 0.29 | 0.35 ± 0.32 | 0.173 | 1.210 | 0.293 | 1.178 (-1.155, 3.512) | 0.836 | 0.379 | 1.394 (-1.929, 4.718) | 0.897 | 0.362 |
|  | Prevotellaceae | 7.48 ± 14.45 | 13.99 ± 28.71 | 0.123 | 1.53 ± 2.69 | 0.55 ± 1.22 | 0.345 | 0.766 | 0.399 | 2.764 (-4.116, 9.645) | 1.197 | 0.295 | 9.692 (-9.607, 28.991) | 1.410 | 0.258 |
|  | Ruminococcaceae | 11.30 ± 7.97 | 19.76 ± 11.97 | 0.061 | 13.98 ± 8.21 | 16.41 ± 8.85 | 0.494 | 4.305 | 0.060 | 5.442 (-0.272, 11.156) | 0.006 | 0.941 | 0.333 (-9.306, 9.971) | 1.319 | 0.273 |
|  | Streptococcaceae | 0.312 ± 0.356 | 0.190 ± 0.222 | 0.484 | 0.067 ± 0.043 | 0.118 ± 0.083 | 0.148 | 0.188 | 0.672 | -0.036 (-0.215, 0.143) | 2.863 | 0.116 | 0.159 (-0.046, 0.363) | 1.105 | 0.314 |
| Genus | Anaerostipes | 0.109 ± 0.171 | 0.121 ± 0.124 | 0.889 | 0.788 ± 0.870 | 0.440 ± 0.289 | 0.428 | 0.925 | 0.355 | -0.168 (-0.547, 0.212) | 10.160 | 0.008 | -0.499 (-0.840, -0.158) | 1.071 | 0.321 |
|  | Asaccharobacter | 0.015 ± 0.028 | 0.034 ± 0.050 | 0.028 | 0.002 ± 0.003 | 0.007 ± 0.012 | 0.180 | 4.875 | 0.047 | 0.012 (0.000, 0.024) | 1.515 | 0.242 | 0.020 (-0.015, 0.055) | 1.576 | 0.233 |
|  | Bacteroides | 25.82 ± 22.56 | 19.11 ± 20.23 | 0.152 | 50.04 ± 10.21 | 51.80 ± 12.54 | 0.744 | 0.578 | 0.462 | -2.480 (-9.585, 4.626) | 9.711 | 0.009 | -28.451 (-48.344, -8.559) | 1.686 | 0.219 |
|  | Butyricicoccus | 0.091 ± 0.085 | 0.088 ± 0.100 | 0.956 | 0.083 ± 0.093 | 0.030 ± 0.045 | 0.075 | 0.977 | 0.342 | -0.028 (-0.089, 0.034) | 0.842 | 0.377 | 0.033 (-0.046, 0.113) | 0.813 | 0.385 |
|  | Clostridium_IV | 0.100 ± 0.121 | 0.086 ± 0.095 | 0.612 | 0.101 ± 0.088 | 0.134 ± 0.193 | 0.753 | 0.040 | 0.846 | 0.009 (-0.093, 0.111) | 0.239 | 0.634 | -0.025 (-0.135, 0.085) | 0.243 | 0.631 |
|  | Clostridium_XlVa | 1.23 ± 0.81 | 2.72 ± 2.65 | 0.093 | 3.50 ± 2.51 | 3.42 ± 2.01 | 0.905 | 1.026 | 0.331 | 0.702 (-0.808, 2.213) | 2.742 | 0.124 | -1.483 (-3.435, 0.468) | 1.281 | 0.280 |
|  | Clostridium_XlVb | 0.157 ± 0.098 | 0.152 ± 0.096 | 0.767 | 0.186 ± 0.168 | 0.345 ± 0.205 | 0.128 | 3.956 | 0.070 | 0.077 (-0.007, 0.161) | 2.820 | 0.119 | -0.111 (-0.256, 0.033) | 4.535 | 0.055 |
|  | Collinsella | 0.52 ± 1.06 | 2.64 ± 6.09 | 0.028 | 0.13 ± 0.19 | 0.30 ± 0.32 | 0.068 | 1.210 | 0.293 | 1.146 (-1.124, 3.416) | 0.865 | 0.371 | 1.371 (-1.842, 4.585) | 0.877 | 0.367 |
|  | Coprococcus | 0.158 ± 0.285 | 0.330 ± 0.376 | 0.063 | 0.192 ± 0.434 | 0.191 ± 0.367 | 0.715 | 3.414 | 0.089 | 0.085 (-0.015, 0.186) | 0.075 | 0.789 | 0.052 (-0.364, 0.468) | 3.509 | 0.086 |
|  | Fusicatenibacter | 0.208 ± 0.186 | 0.983 ± 0.887 | 0.049 | 0.417 ± 0.621 | 0.366 ± 0.408 | 0.753 | 3.071 | 0.105 | 0.362 (-0.088, 0.812) | 0.686 | 0.424 | 0.204 (-0.333, 0.741) | 4.005 | 0.069 |
|  | Megasphaera | 0.02 ± 0.03 | 0.04 ± 0.05 | 0.674 | 1.17 ± 1.85 | 0.16 ± 0.19 | 0.249 | 2.947 | 0.112 | -0.498 (-1.129, 0.134) | 3.227 | 0.098 | -0.637 (-1.410, 0.136) | 3.203 | 0.099 |
|  | Prevotella | 7.43 ± 14.34 | 13.64 ± 27.74 | 0.123 | 1.53 ± 2.69 | 0.54 ± 1.22 | 0.345 | 0.765 | 0.399 | 2.608 (-3.888, 9.104) | 1.209 | 0.293 | 9.946 (-9.323, 28.314) | 1.459 | 0.250 |
|  | Ruminococcus2 | 0.225 ± 0.193 | 0.224 ± 0.310 | 0.575 | 0.307 ± 0.336 | 0.153 ± 0.173 | 0.102 | 2.179 | 0.166 | -0.077 (-0.192, 0.037) | 0.002 | 0.967 | -0.006 (-0.292, 0.281) | 2.138 | 0.169 |
|  | Sutterella | 0.192 ± 0.486 | 0.158 ± 0.385 | 0.593 | 0.272 ± 0.463 | 0.448 ± 0.695 | 0.068 | 1.550 | 0.237 | 0.071 (-0.053, 0.196) | 0.475 | 0.504 | -0.185 (-0.768, 0.399) | 3.386 | 0.091 |

Baseline: pre-exercise; After intervention: after 8-week exercise. Paired samples t-test was used to indicated intra-group differences of gut microbiota for data with normal distributions or Wilcoxon signed-ranks test for data with non-normal distributions. The group × time interaction of changes in gut microbiota composition was estimated by repeated-measures analysis of variance after 8-week exercise training
